# Transthoracic Echocardiographic Findings of Stanford Type A Aortic Dissection: A Case Report

**DOI:** 10.7759/cureus.6207

**Published:** 2019-11-20

**Authors:** Alexandra Craen, Javier Rosario, Kendra Amico, Mihir Tak, Latha Ganti

**Affiliations:** 1 Emergency Medicine, University of Central Florida College of Medicine, Orlando, USA; 2 Emergency Medicine, Envision Physician Services, Orlando, USA

**Keywords:** aortic dissection, ultrasound

## Abstract

Aortic dissection is a well-known, but relatively uncommon diagnosis in the emergency department (ED). With a mortality rate as high as 30 percent, it is important to be able to diagnose quickly and accurately. Definitive diagnosis with imaging studies such as computed tomography angiogram (CTA) can be expensive and time-consuming and may not always be available in the community. Herein, we discuss a case of a 59-year-old man presenting with severe chest pain, hypotension, and bradycardia who was diagnosed with aortic dissection first by bedside ultrasound. This expedited the CTA and a cardiothoracic surgery consult, leading to a successful emergent aortic repair.

## Introduction

Aortic dissection is a relatively rare emergency with a high mortality rate, making early diagnosis and treatment essential in the emergency department (ED). The incidence of aortic dissection is around three per 100,000 with a mortality rate of 25 to 30 percent [1-3]. It is most commonly seen in men in their 60s, and hypertension is the most common predisposing factor [3]. It can have variable presentations that make diagnosis difficult. Sudden-onset tearing chest pain, mediastinal or aortic widening on chest radiography, and pulse or blood pressure differentials were found to be independent predictors [4]. The Stanford system is the most used anatomic classification system for aortic dissections. Any dissection involving the ascending aorta is considered type A and all others are type B. Type A dissections are a surgical emergency and type B dissections are usually managed medically [3].

## Case presentation

A 59-year-old male with a past medical history of tobacco and marijuana use, chronic back pain not on narcotics, and untreated hypertension and dyslipidemia was transferred from an outside hospital for further workup after presenting there with acute substernal chest pain radiating to his anterior neck. He was found to be hypotensive and bradycardic with a blood pressure of 70s/40s and a heart rate in the 40s. He was given atropine in route and on arrival received a normal saline bolus, morphine, ondansetron, and lorazepam for his symptoms. The electrocardiogram showed sinus bradycardia with no ST-segment elevations. Chest radiography showed a mildly enlarged cardiac silhouette with no acute abnormalities. Labs were significant for a negative troponin and an elevated D dimer to 35 mg/L FEU. He was transferred to our facility because their CT scan was not functioning.

**Figure 1:**
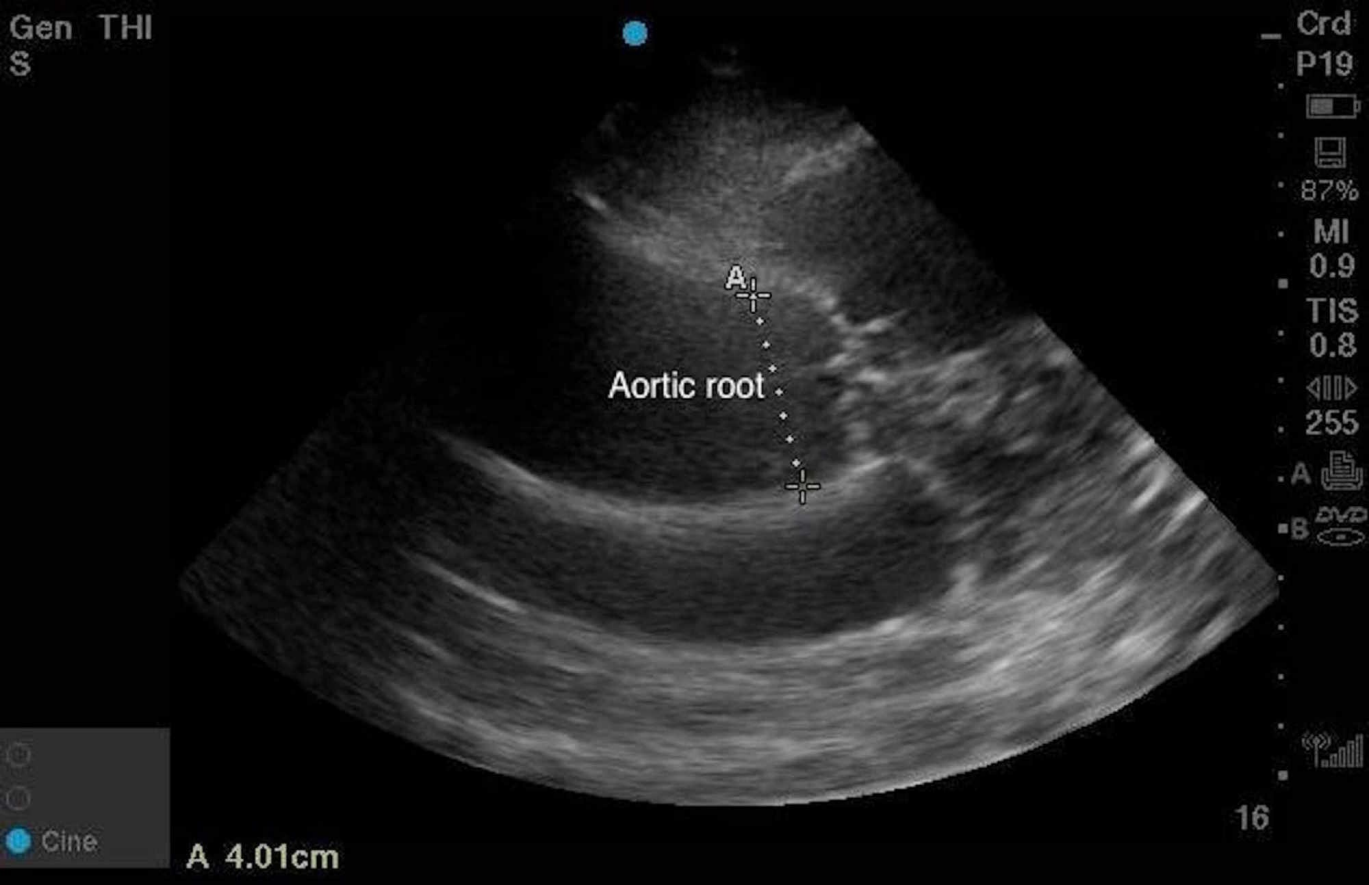
Ultrasound image of dilated aortic root

**Figure 2:**
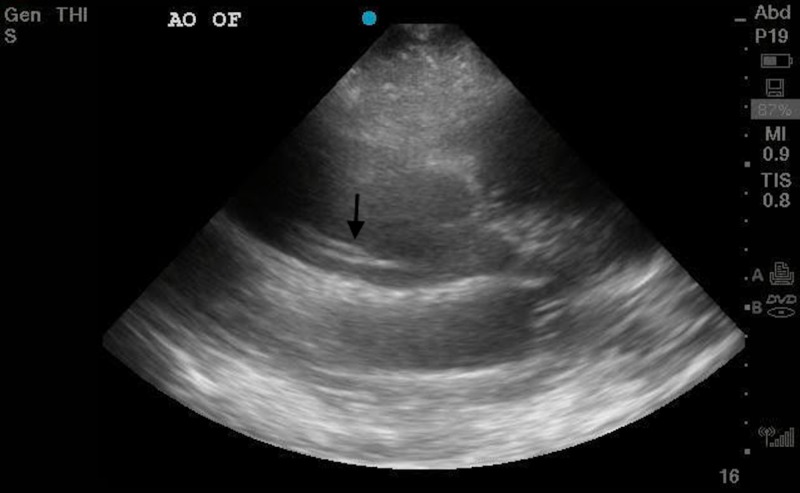
Ultrasound image of an intimal flap by the aortic root

On arrival, the patient had a blood pressure of 114/45 with a heart rate of 54 bpm. He continued to have constant chest pain. On physical exam, he had normal heart sounds and equal radial and femoral pulses. His blood pressure could not initially be obtained on the right upper extremity, but after being taken manually was similar to the left. His electrocardiogram showed low voltage sinus bradycardia, and so a bedside transthoracic echo (TTE) was done. This showed a dilated aortic root with an intimal flap suggestive of aortic dissection (Figures 1, 2).

**Figure 3:**
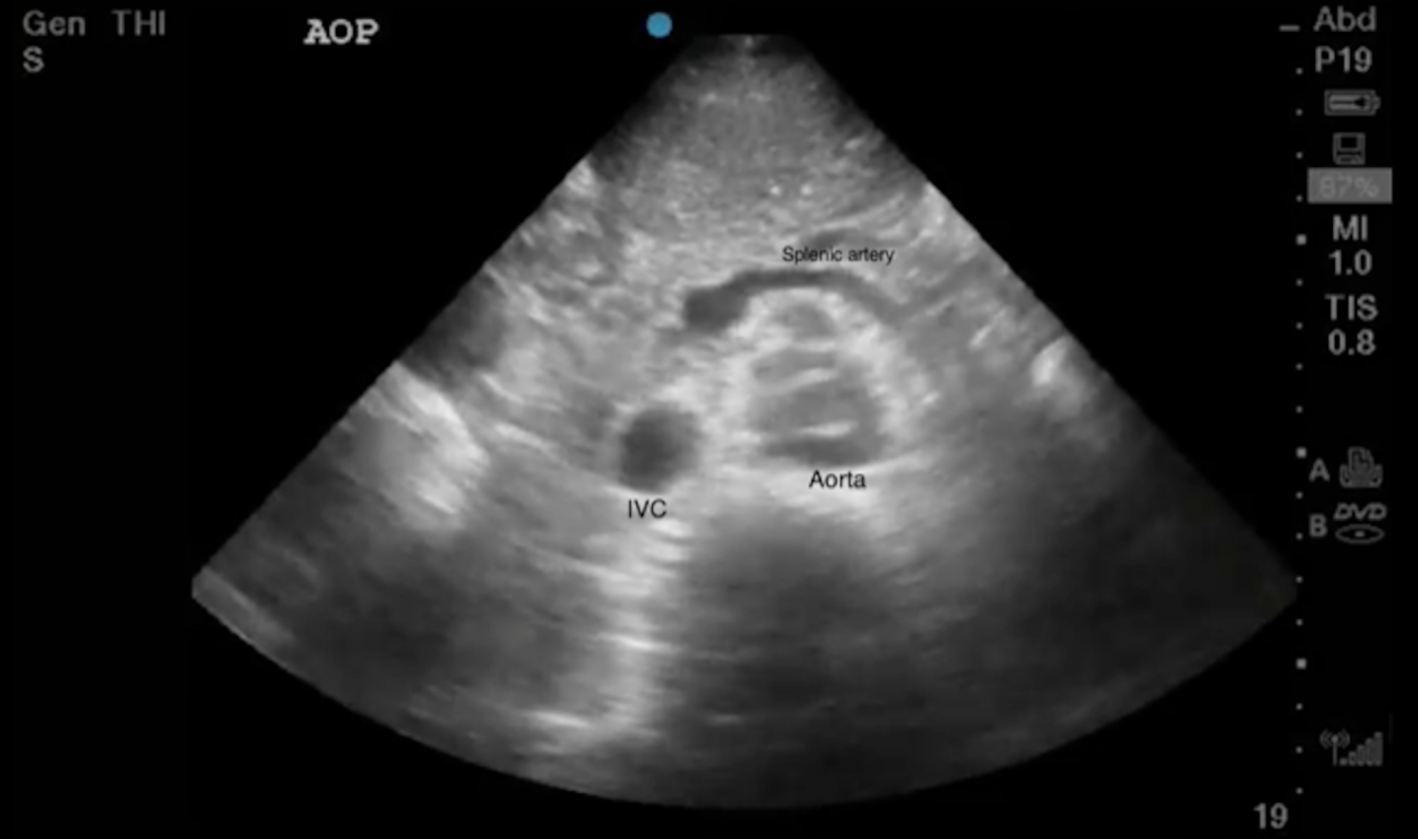
Transverse ultrasound image of intimal flaps in the aorta from a dissection

**Figure 4:**
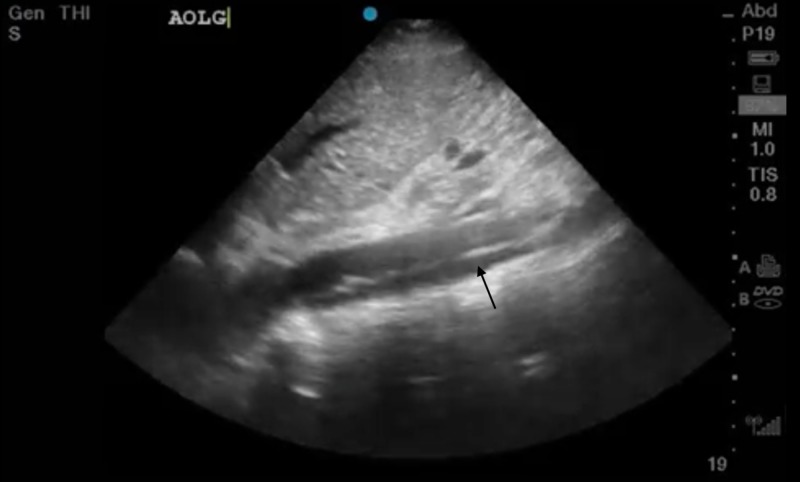
Longitudinal ultrasound image of intimal flap in abdominal aorta

The abdominal aorta was then evaluated and also showed a flap extending to the renal arteries (Figures 3, 4).

No effusions or right heart strain was noted. The patient was taken emergently to CT scan, and the results were discussed with the radiologist over the phone immediately. This information was then relayed to the cardiothoracic surgeon.

**Figure 5:**
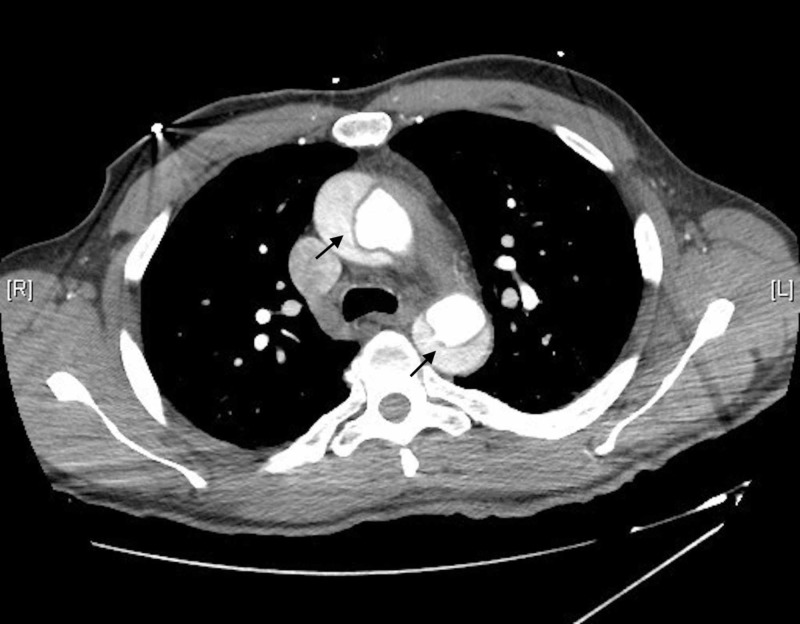
CT image of intimal flaps in ascending and descending aorta CT, computed tomography

**Figure 6:**
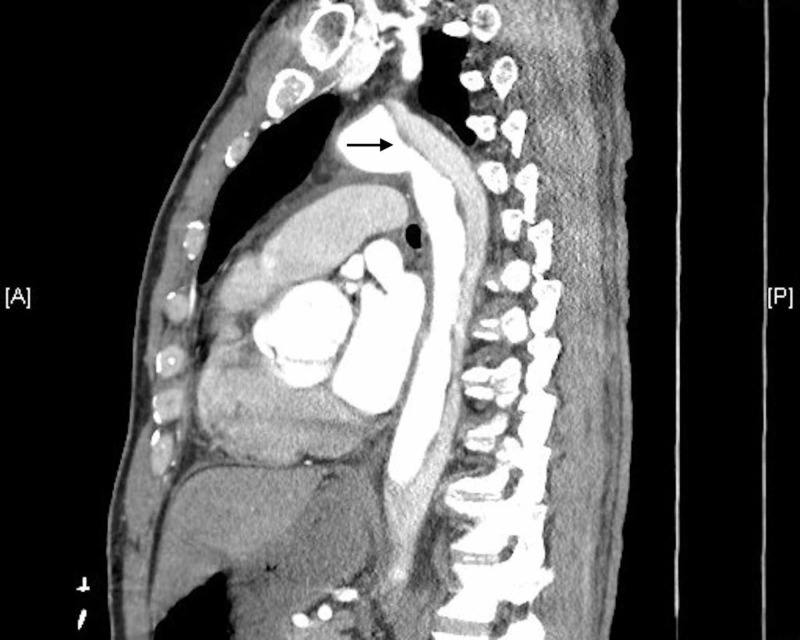
Sagittal CT view of aortic dissection CT, computed tomography

The CTA chest and abdomen/pelvis showed a Stanford type A aortic dissection (Figures 5, 6).

**Figure 7:**
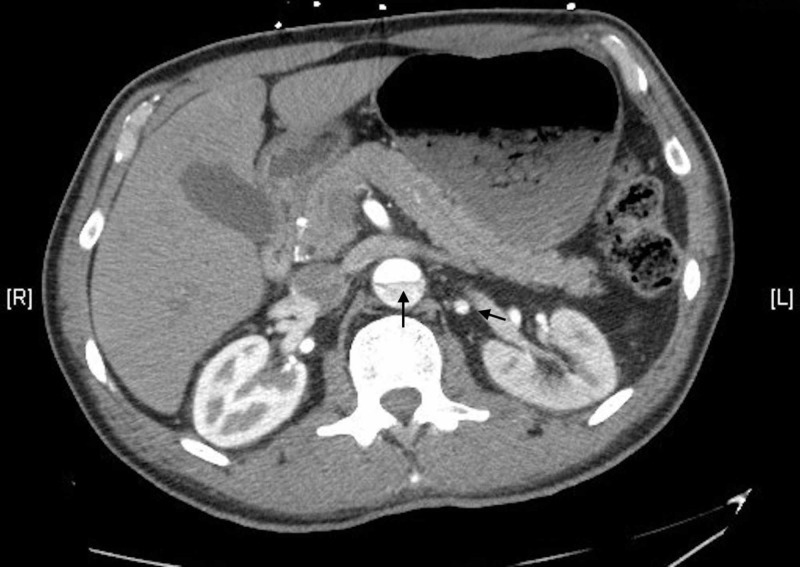
CT image of dissection extending to distal abdominal aorta and iliac artery CT, computed tomography

An intimal flap began at the aortic root and extended throughout the ascending and descending thoracic aorta into the bilateral common iliac arteries (Figure 7).

The dissection also extended into the right brachiocephalic artery and proximal right common carotid artery. The ascending thoracic aortic aneurysm measured up to 4.8 cm, and the proximal descending thoracic aortic aneurysm measured up to 3.9 cm.

The cardiothoracic surgeon immediately reviewed the images and took the patient for an emergent aortic dissection repair with a 32-mm hemashield graft placed and resuspension of the aortic valve. He was discharged 10 days later to a cardiac rehabilitation facility after an unremarkable postoperative course.

## Discussion

The high morbidity and mortality of aortic dissections make methods of the timely diagnosis of this emergency an important consideration. The gold standard imaging for the diagnosis of an acute aortic syndrome (AAS) remains imaging with CTA, magnetic resonance angiography (MRA), or transesophageal echocardiography (TEE) with CTA the study of choice in the ED [5]. D-dimer testing has been studied as a way to rule out AAS when there is low clinical suspicion using the aortic dissection detection (ADD) risk score, but it has not been externally validated [6]. Our patient had a significantly elevated d-dimer that necessitated additional chest imaging. Given the high clinical suspicion, CTA could have been performed immediately.

The initial facility did not have the ability to perform CTA when the patient presented. Bedside TTE is an important diagnostic tool for resource-poor facilities in patients with chest pain. Our patient presented with hypotension, and a bedside TTE can quickly evaluate for not only pericardial effusions and heart strain but also aortic dissection as with our patient. The focus of emergency management of aortic dissection is traditionally heart rate and blood pressure control; however, hypertension is seen acutely in only 25 to 35 percent of type A dissections with hypotension present in 25 percent [3]. Causes could include acute aortic valve regurgitation, cardiac tamponade, acute myocardial ischemia, or aortic rupture and exsanguination [3,7]. Bedside ultrasonography is a valuable tool to identify and intervene in these complications [5,8].

There are few quality studies addressing the use of bedside TTE by trained emergency physicians at this time. Most recommend its use as a rapid triage tool [5,7,9]. It can also be considered in patients with renal dysfunction or contrast allergies [7]. The American College of Emergency Physicians (ACEP) clinical policy for suspected acute nontraumatic thoracic aortic aneurysm did not recommend using bedside TTE to establish a definitive diagnosis as a level B recommendation, but they do recommend surgical consultation or transfer to a higher level of care if the TTE is suggestive of aortic dissection as a level C recommendation [10]. Further research is needed to determine the accuracy and limitations of bedside TTE.

## Conclusions

Acute aortic dissections are true emergencies where rapid intervention is necessary. The diagnosis is complicated by variations in presentation, the potentially time-consuming process of imaging, or serious contrast allergies. A suggestive bedside TTE can identify emergent complications and expedite further imaging, consultation, and transfer as needed. With further development of emergency physician ultrasound skills and research, its utilization could become a more fundamental part of aortic dissection diagnosis and management.
